# Fluid flow to mimic organ function in 3D *in vitro* models

**DOI:** 10.1063/5.0146000

**Published:** 2023-08-04

**Authors:** Yago Juste-Lanas, Silvia Hervas-Raluy, José Manuel García-Aznar, Alejandra González-Loyola

**Affiliations:** 1 Department of Mechanical Engineering, Engineering Research Institute of Aragón (I3A), University of Zaragoza, Zaragoza, Spain; 2 Department of Biochemistry and Molecular and Cellular Biology, University of Zaragoza, Zaragoza, Spain; 3 Aragon Health Research Institute (IIS Aragón), Zaragoza, Spain

## Abstract

Many different strategies can be found in the literature to model organ physiology, tissue functionality, and disease *in vitro*; however, most of these models lack the physiological fluid dynamics present *in vivo*. Here, we highlight the importance of fluid flow for tissue homeostasis, specifically in vessels, other lumen structures, and interstitium, to point out the need of perfusion in current 3D *in vitro* models. Importantly, the advantages and limitations of the different current experimental fluid-flow setups are discussed. Finally, we shed light on current challenges and future focus of fluid flow models applied to the newest bioengineering state-of-the-art platforms, such as organoids and organ-on-a-chip, as the most sophisticated and physiological preclinical platforms.

## I. INTRODUCTION

Biological models are continuously evolving, and three-dimensional (3D) *in vitro* cell cultures have been consolidated as an adequate alternative to overcome the drawbacks of classical biological models. On one hand, 3D *in vitro* cultures improve the low levels of biological complexity and realism of two-dimensional (2D) *in vitro* models.^1,2^ On the other hand, they avoid the ethical implications of *in vivo* models, allowing a robust control of the experimental parameters, reducing time and cost of experiments,^2–4^ while closely representing the human pathophysiology.^5,6^

The extracellular matrix (ECM), essential physical scaffolding that provides key biochemical and biomechanical cues,^5^ is commonly represented in 3D *in vitro* models using hydrogels.^7^ These gels accurately mimic the biological features of the ECM, and they comprise the best option for engineering tissue models in microfluidic devices and bioreactors, including organoids and organ-on-a-chip systems.^7,8^ Natural materials like collagen,^9–11^ matrigel,^9,12–19^ or fibrin^17^ are widely used, but other synthetic materials, with less degradability and further stability, like polyethylene glycol (PEG)^20^ are also utilized. In addition, combined materials are employed to form hydrogels with different mechanical properties such as mixtures of gelatin–fibrin,^21^ matrigel–fibrin,^17^ matrigel–fibrin–gelatin.^22^

Importantly, fluid flow has an important role in organ morphogenesis, homeostasis, and pathogenesis.^23,24^ Flow induces cell mechanical stimulation in luminal structures and the interstitial space,^25–27^ and it transports fundamental nutrients and signaling molecules,^24^ and it may alter the ECM.^28–30^ Many 2D systems have incorporated fluid flow component in their models; however, most 3D cell cultures (i.e., ECM-based cell cultures) still lack the physiological fluid dynamism present *in vivo* (supplementary material, Fig. 1).

Fluid flow stimulates cells mechanically by inducing shear stress (tangential to cell surface, along the direction of the flow) and pressure stress (normal to the cell surface)^31,32^ (see Glossary Box). These stresses participate in the regulation of cell proliferation, quiescence, differentiation, or migration processes.^33^

In vessels, flow sensing plays a critical role in development and maintenance *via* multiple pathways.^34–36^ An adequate continuous luminal fluid shear stress (FSS) enhances endothelial cells' (ECs) physiological behavior and cell differentiation,^37^ while abnormal FSS (generated by altered flow rates and/or flow directions) can result in EC sprouting and endothelial-to-mesenchymal transition (EMT), vascular dysfunction, or atherosclerosis.^34,38–40^ In addition, luminal fluid pressure produces strain on the endoluminal surface, which, among other effects, regulates the thickness of the vessels, i.e., by increasing smooth muscle cell (SMC) hypertrophy and the content of collagen and elastin.^31,41^

**FIG. 1.**
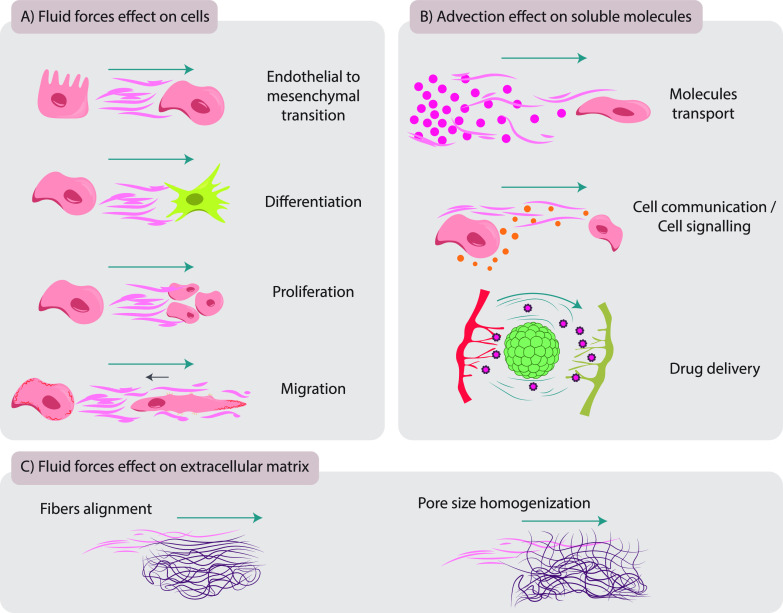
The impact of fluid forces on cells, soluble molecules, and extracellular matrix (ECM). (a) The effect of fluid forces on cells affecting endothelial–mesenchymal transition, differentiation, proliferation, and migration. (b) Advection effect on soluble molecules, regulating nutrients transport, generating chemical gradients, and controlling cell communication/cell signaling and drug delivery (fluid flow is represented by blue lines). (c) The effect of fluid forces on the ECM, favoring ECM fibers' alignment and pore size homogenization.

In the interstitium, fluid forces regulate the activity of fibroblasts (FBs), the most common cell type of connective tissue. Interstitial fluid forces activate/differentiate FBs, augmenting their migration and fibrogenesis-related features.^27,28^ They increase FB adhesion to the ECM in exposed areas, resulting in cell polarization, actin accumulation, and membrane protrusion in the up-stream direction^27^ [Fig. 1(a)].

The transport of molecules in a biological fluid, mainly regulated by diffusion (random motion of molecules) and advection (molecules' movement by the fluid's bulk motion), is also affected by flow (see Glossary Box).^25,33,42^ Flow advection directly impacts on nutrients distribution, cell communication, and drug delivery [Fig. 1(b)]. Thus, the undesired results of the lack of advection can be accentuated in large-scale *in vitro* cell cultures, where tissue thickness hinders the transport of nutrients and gasses.^43^ In addition, as diffusion is reduced with increasing molecular size,^44^ the advection generated by fluid flow is especially relevant for the transport of large molecules like antibodies, nanoparticles, or exosomes, carriers of advanced medicines that harbor a large size in comparison with previous drugs.^45^

Glossary Box:

The following are the types of stresses induced by fluid flow:

•   Shear stress (τ): mechanical force per area exerted tangential to the cell surface, along the direction of flow.
•   Pressure stress (p): mechanical force per area induced perpendicular to the cell surface

Transport of molecules in a fluid is mainly regulated by the following:

•   Diffusion: net movement of solutes from a region of higher solute concentration to a region of lower solute concentration due to the random motion of the solute and water molecules.
•   Advection: molecules movement/transport by a fluid bulk motion.

Other key elements of fluid dynamics in microfluidic devices are as follows:

•   Dynamic viscosity or absolute viscosity (μ): refers to internal resistance that a fluid exerts to flow.
•   Kinematic viscosity (η): refers to the ratio of dynamic viscosity to density.
•   ECM permeability (K): refers to the resistance of a tissue or hydrogel to let the fluid flow through it.

The above-described variables are related in Newtonian fluids by the following equations:

$$\mathrm{In}\;\text{luminal}\;\text{flow},\;Q=\frac{\Delta p}{R_{h}};\;\mathrm{in}\;\text{interstitial}\;\text{flow},\;Q=\Delta p\cdot k.$$

$$\begin{array}{c} To\;\text{calculate}\;\text{shear}\;\text{stress}\;(\mathrm{in}\;\text{both}\;\text{cases}):\tau =\mu \cdot \frac{\mathrm{dU}}{dy}\\ (y=\text{distance}\;\text{between}\;\text{the}\;\text{surfaces};\;U=\text{velocity}\;\mathrm{of}\;\text{the}\;\text{fluid}). \end{array}$$

Therefore, in a defined section of a vessel or tissue, and a given flow (Q), the viscosity of a fluid (μ)will directly regulate the shear stress (τ). To achieve the same level of flow (Q) with a highly viscous fluid, the system will require a higher pressure drop (Δp). The permeability of the tissue (k) or the hydraulic resistance of the vessel (Rh) will determine the required pressure drop (Δp).

Conversely, in a defined section of a vessel or tissue, and a given pressure drop (Δp), a high viscosity of the fluid will reduce the flow (Q), also conditioned by the permeability of the tissue (k) or the hydraulic resistance of the vessel (Rh).

Most biological fluids show non-Newtonian behavior (the viscosity is not constant, it is dependent on the shear rate); thus, the above-explained equations are used as an approximation, given the difficulty of studying non-Newtonian behavior.

Regarding the interstitial fluid forces' effect on the ECM, although they have not been studied independently of cells *in vivo*, *in vitro* experiments show that fluid forces may promote the homogenization of the pore size and the alignment of ECM fibers ^28–30^ [Fig. 1(c)], as will be discussed in Sec. II.

This work is organized as follows. First, we describe the role of fluid flow for tissue development, homeostasis, and disease, specifically in vessels, other lumen structures, and interstitium, to highlight the need of perfusable and agitation platforms in current *in vitro* 3D models (Sec. II). Next, the advantages and limitations of different experimental fluid flow approaches in ECM-containing microfluidic devices and other bioreactors are discussed (Sec. III). Then, we shed light on fluid flow models applied to the newest bioengineering applications, such as, organoids and organ-on-a-chip, as the most sophisticated and physiological preclinical platforms, with the aim of reaching personalized and regenerative medicine (Sec. IV). Finally, we discuss current challenges and future perspectives in the field (Sec. V).

## II. FLUID FLOW IN DEVELOPMENT, HOMEOSTASIS, AND DISEASE: ESSENTIALS OF PERFUSED 3D *IN VITRO* MODELS

As mentioned before, fluid flow is a key regulator for the maintenance of cellular homeostasis, soluble molecules' transport, and ECM structure. In this section, we highlight the importance of fluid forces across the human body to regulate tissue development, homeostasis, and in pathological conditions such as cardiovascular diseases or cancer. We will also comment on the *in vitro* models used to mimic blood and lymphatic vasculature and other lumen-endothelialized and epithelialized structures.

### A. Relevance of flow in blood vasculature

Blood endothelial cells (BECs) sense flow mechanical forces and transduce the signal to different biological outcomes in pivotal processes that will be discussed through this section, such as vascular development, homeostasis, or various disease states.^37,46,47^

During embryonic development, blood vessels are formed first through vasculogenesis and later through angiogenesis.^48^ Then, vascular network growth and remodeling takes place when blood circulation has already begun, and the endothelium is exposed to fluid mechanical forces such as shear stress, circumferential stress, and axial stress. Intraluminal shear stress is the force parallel to the tissue surface of the endothelium that arises due to flow of a viscous fluid and depends on the flow rate, viscosity of the blood, as well as on the geometry of the vessel. Circumferential stress (force tangential to the vessel wall) and axial stress (along the longitudinal axis) are governed by the intraluminal pressure. These forces together with transendothelial blood flow (across endothelium) are needed for the expansion of the primary vascular plexus (sprouting angiogenesis).^39,49^

Adult homeostatic blood vessels (BVs) are formed by ECs lining the inner lumen, which are covered by a basal membrane and pericytes in capillaries, together with smooth muscle cells and adventitia in the case of arteries and veins. The intraluminal shear stress and stretch generated by luminal and transendothelial blood flow, together with intraluminal pressure, govern cell morphology, proliferation, protein expression, and the ability of ECs to attract monocytes.^50^ Additionally, these forces regulate vessel tone by inducing adaptive dilation of the vessel wall through the activation of the ion channel Piezo1 in ECs and the release of nitric oxide.^51^

Cardiovascular diseases are the main cause of death globally. One of the most common cardiovascular pathologies is BV occlusion due to atherosclerosis (lipidic plaque formation in arteries) or thrombosis.^52,53^ Disturbed blood shear stress in vessel branch points or bifurcations is known to induce EC proliferation and activation of inflammatory pathways, thus predisposing these sites to atherosclerosis.^51^ Turbulent flow shear stress is atherogenic as a result of Piezo1-dependent stimulation of the NF-kB pathway in ECs.^54,55^ Conversely, the etiology of some vascular malformations such as arteriosus venous malformations is associated to altered blood flow due to vessel enlargement.^56^

#### 1. 3D *in vitro* models to mimic blood vasculature

To investigate vessel development, homeostasis, and disease states, tissue engineering approaches have been developed. Tissue engineering models have tried to implement functional vasculature by generating perfusable vascular units.^57,58^ To this end, preformed vessels can be developed in polydimethylsiloxane (PDMS) by soft lithography^59^ or microfabricated in plastic^58^ or hydrogel.^60,61^ They can also be produced in hydrogels by sacrificial molds^62,63^ or laser ablation.^64^

First, with the aim of investigating angiogenesis, different strategies have been used such as ECs monolayers^65,66^ or endothelialized microvessels,^67^ where sprouting or angiogenesis processes occur in response to the stimulation with the vascular endothelial growth factor (VEGF).^68–70^ As discussed above, since the mechanical stimuli exerted by blood flow is a required physiological factor for new vessels formation,^71^ recent *in vitro* vascularized models aiming to study angiogenesis recapitulate flow forces to generate more reliable results.^65,72,73^ When this process starts, ECs begin to remodel the ECM by degrading the matrix *via* the release of metalloproteinases (MMPs),^74,75^ which allow EC migration and microvascular network formation.^68,76^ As an example, Kim *et al.*^57^ developed a complete microvascular network of ECs, where they studied EC interaction with pericytes. More recently, 3D bioprinting methods have been used for precise positioning of ECs^77–79^ to decipher the mechanisms behind vascular morphogenesis processes.^80,81^

**FIG. 2.**
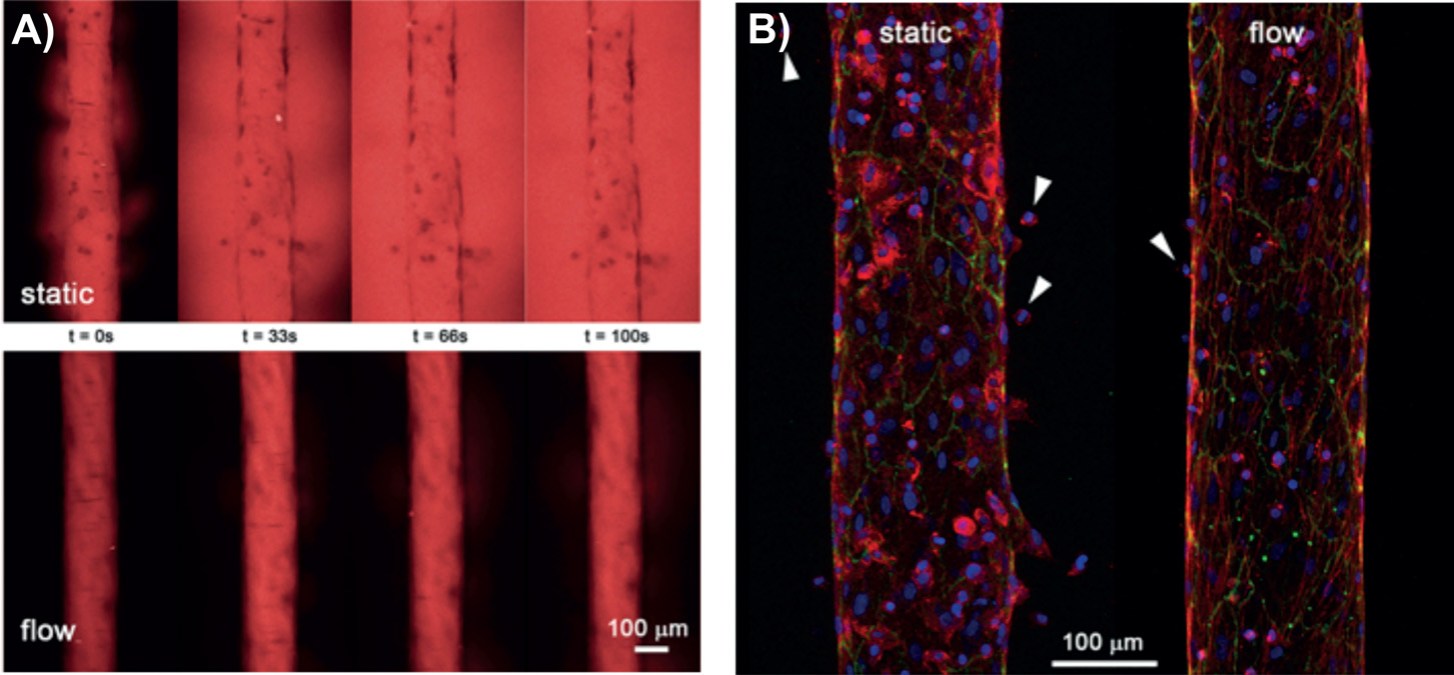
Effect of FSS in blood vessel structure and function. (a) 70 kDa red-fluorescent dextran permeability assay. Vessels generated under flow show high endothelial integrity and reduced permeability compared to vessels under static condition. (b) Reduced monocyte extravasation in vessels formed under flow. Monocytes are indicated with white arrows. Endothelial vessels are stained for F-actin(red), VE-cadherin (green), and DAPI (blue). Adapted with permission from Pérez-Rodríguez *et al.*, J. Biomicrofluidics **15**, 0541012 (2021). Copyright 2021 Authors, licensed under a Creative Common Attribution (CC BY) license.^67^

Second, to mimic homeostatic BVs *in vitro*, lumenized constructs covered with ECs are made by an endothelialization process. The structure, in contact with an ECM, may also include mural cells to recapitulate the *in vivo* physiology of BVs.^82^ To achieve flow stimuli in these structures, different perfusion systems including rockers,^72^ fluid columns,^65^ or peristaltic pumps^73^ have been designed, according to the purpose of the investigation (as discussed in Sec. III). These models have served to study physiological events occurring *in vivo,* highlighting the role of FSS to maintain endothelial cell integrity, while reducing vessel permeability and immune cell extravasation, compared to vessels under static conditions (Fig. 2).^67^

Third, perfused 3D *in vitro* models can be effective tools to gain insight into the mechanisms driving the progression of different pathologies. For instance, to study cardiovascular diseases such as atherosclerosis or thrombosis, a microvascular network formation with flow path can be relatively easily implemented in microfluidic devices.^83^ However, to accurately simulate pathologies in arteries, all relevant stresses and strains should be incorporated into a single setup, and designing such a model is a complex task that requires careful consideration. Capturing the full complexity of *in vivo* flow is an extremely challenging task *in vitro*. Therefore, it is crucial to carefully consider the balance between building a system that is limited by over-complexity versus carefully tuning specific flow parameters.

### B. Relevance of flow in lymphatic vasculature

The lymphatic system, which is precisely coordinated with the cardiovascular system, transports fluid and cells from the interstitial space to lymph nodes and, then, to the blood circulation.^84^ Lymphatic endothelial cells (LECs) like BECs are able to mechanosense fluid flow, which is crucial for the development, maintenance, and disease of the lymphatic system, as it will be discussed through this subsection.

Lymphatic vascular development starts by LECs' transdifferentiation from venous ECs (in most cases, though alternative sources have been observed).^85^ LEC progenitors coalesce and expand to form primitive plexus. Then, under pressure gradient, fluid is transferred from the interstitial space into the lumen of the lymphatic plexus. This process stretches LECs and leads to the expansion of the lymphatic plexus. Mechanical stretching activates integrin ß1, which enhances VEGF-C-dependent phosphorylation of VEGF receptor 3 (R-3). The activation of this signaling pathway potentiates LEC proliferation and expansion of the network.^86^ Additionally, laminar shear stress induced by intraluminal flow inhibits NOTCH1 and induces Ca2+ signaling and the transcription factors KLF2/4 that promote LEC proliferation and sprouting^87–89^ till a more mature network consisting in capillaries and collecting lymphatic vessels (LVs) is formed. Indeed, for the formation of collecting LVs and valves, lymph flow sensing by LECs is needed in areas of flow recirculation.^37,90,91^

Adult homeostatic lymphatic capillaries are formed by LECs lining the inner lumen with discontinuous “button- like” junctions, discontinuous basement membrane, and no supporting mural cells to ensure the absorption of liquid from the interstitium. Whereas adult collecting LVs are formed by LECs with a continuous basement membrane, continuous zipper-like junctions, smooth muscle cell coverage, and intraluminal valves to transport the lymph unidirectionally.^92^ As transendothelial flow in capillary LVs has been related to junctional maturation,^93^ LV valve maintenance has been shown to be dependent on luminal FSS.^94^ Studies conducted on cultured BECs and LECs have demonstrated that VEGFR-3 is involved in regulating the setpoint of FSS, which is the preferred range of shear stress that induces physiological endothelial responses to flow. Therefore, VEGFR-3 can alter cell alignment or suppression of inflammation, and, thus, contribute to the maintenance of vascular homeostasis.^95^

Dysfunction of the lymphatic system has been related to devastating diseases, such as lymphedema, inflammation (e.g., Crohn's disease), tumor metastasis, obesity, glaucoma, cardiovascular, or neurovascular pathologies.^96^ In these pathologies, defective LVs induce lymph backflow, which is related with many of the outcomes of the diseases. As an example, in the case of lymphedema, defective lymph drainage frequently leads to tissue fibrosis due to lymph stasis together with fat deposition and local immunodeficiency, caused by the abnormal local chronic inflammatory response, which, in turn, increases susceptibility to infections.^97^ Additionally, an abnormal cardiac lymph flow has been shown to induce cardiac edema and inflammation, thus affecting heart fluid balance and immune surveillance, which is partially reduced when inducing lymphangiogenesis.^98^

#### 1. 3D *in vitro* models to mimic lymphatic vasculature

In the last decade, some engineering studies modeling perfused LVs *in vitro* have been published. Osaki *et al.* modeled LVs to study lymphangiogenesis and its interaction with angiogenesis^73^ using molded microchannels within ECM collagen gel, perfused with a peristaltic pump. Moreover, studying LVs in microfluidic platforms opens new avenues for understanding organ homeostasis and pathology.^42^ To mimic the transport kinetics of biomolecules and drugs, Zhang and colleagues used bioprinting techniques with very precise bioink flow rate to construct the wall thicknesses of an end-blinded lymphatic capillary next to a blood vessel.^99^ Though this system lacks cell lining and, thus, active transport, the model could provide insight on how the transport of biomolecules happens by diffusion. On the other hand, since LVs are one of the preferential routes for metastases, Lee *et al.*^100^ studied, *via* syringe pumps, how FSS through a cylindrical lymphatic channel modifies cancer cell motility. Also, breast cancer models with tubular LVs have been generated to study the effect of ECM density on LV morphology, growth, cytokine secretion, and barrier function.^101^

An additional challenge to increase the biological relevance of microfluidic devices is using more realistic perfusate fluids. The commonly used synthetic (i.e., commercial) cell culture media do not completely correlate with the biochemical composition of the fluids to which cells are exposed *in vivo,* which can alter the gene expression and relevance of *in vitro* models.^102^^,^^103^ Furthermore, the biophysical properties of the synthetic media, widely used in perfused *in vitro* models,^45^^,^^104^ also affect cell behavior.^105^ Commercial media have reduced viscosity (∼0.8 cP or mPa·s)^45^^,^^106^ in comparison with blood (∼3.4 cP) or lymph viscosity (∼1.8 cP),^45^ which lowers the resultant shear stress and pressure exerted on cells (in flow-controlled experiments).^106^

### C. Other lumen-endothelialized or epithelialized structures

Our body also contains other tubular structures exposed to fluid flow and shear stress, such as renal convoluted proximal tubules,^107,108^ renal collecting ducts,^109^ liver sinusoids,^110^ or the gut.^111^ Particularly, their morphology (i.e., cytoskeleton organization, microvilli formation) and function (i.e., cubilin/megalin, albumin, or drug transport),^32^ as well as their differentiation, are affected by fluid mechanical forces, and 2D static models cannot predict tissue functions that arise in perfused 3D geometries.^107,109^ For example, FSS in the kidney nephron is thought to vary from ∼1.0 dyne/cm^2^ in the proximal tubule to <0.5 dyne/cm^2^ in the collecting duct, while pressure can range between ∼13 mmHg in the proximal tubule to <7 mmHg in the collecting duct.^112^

#### 1. 3D *in vitro* models to mimic other lumen or epithelized structures

To engineer the above-mentioned tubular structures *in vitro*, close-loop perfused 3D models of renal tubules can be built as conduits together with a blood vessel conduit, allowing advanced functional studies with precise tubular-vascular metabolite exchange.^113^ Similarly, perfusion systems can be incorporated to 3D bile ducts to functionally activate cholangiocytes,^114^ or to intestines to study their epithelial barrier integrity^115^ and prolong tissue lifespan by several weeks.^116^

Different to hydrogel ECM, hydrogel-coated membranes are widely used to simplify the study of flow-induced dynamic lumen–epithelial interactions and facilitate the generation of more complex structures. This is the case of liver sinusoids, the basic structural unit of the liver (composed of liver sinusoidal ECs, holding Kupffer cell, stellate cells, and hepatocytes^117^), whose functionality is enhanced by flow shear stress^118^ (*in vitro* modeling of liver sinusoids is fully reviewed here^119^). Hydrogel-coated membranes are, as well, used in intestinal crypt-villi structures ^120,121^ and microbial-gut-vessel interactions,^122^ also reviewed elsewhere.^123–126^ However, these membranes, often made of silicone-like PDMS^104,127^ or polycarbonate and polyester,^128,129^ poorly mimic the biological and physicochemical properties of the basal membrane and interstitium. This is important to be considered in terms of tissue stiffness,^127^ soluble molecules transport,^128^ and cell viability and function.^128^ Nevertheless, as hydrogel-based membranes lack durability,^129^ synthetic materials, usually coated with a collagen or Matrigel layer to enhance cell adhesion,^104^ are still used because of their good visualization, manipulation, and biocompatibility.^104,129^

### D. Relevance of interstitial fluid flow (IFF) and transendothelial flow

In nearly all tissues, plasma leaks out of blood capillaries, flows through the interstitium, and drains into LVs, where it passes through lymph nodes before being returned to the blood circulation. The plasma filtrate that flows between BVs, the interstitium, and LVs is known as interstitial fluid. The interstitial fluid flow (IFF) originates from blood vessels' transendothelial flow, driven by the difference of hydrostatic and osmotic pressure (termed Starling forces) between the blood vessels and the interstitium.^130^ The resultant transendothelial and IFF highly contribute to tissue development, homeostasis, and pathogenesis, physiologically reaching up to 20% of the body mass,^131^ moving at 0.1–2.0 *μ*m/s^132^ and generating a shear stress around 0.1 dynes/cm^2^.^133^

The advection of transendothelial and IFF provides nutrient renewal to tissues. Flow regulates *via* shear stress and the transcellular stress gradient generated, together with other factors, the migration of cells such as FBs, ECs, and mesenchymal stem cells, and specifically, the behavior of focal adhesion kinases (FAKs) and integrins,^134–138^ as well as MMPs.^134,139^ Furthermore, interstitial fluid forces can directly impact the alignment of ECM fibers, although not homogeneously across matrices. Current evidence suggests that dense and crosslinked collagen gels ^29^ are unaffected by IFF, while dense but less crosslinked collagen gels ^29^ and fibrin gels ^140^ may slightly align their fibers in the direction of the flow. Matrices of reduced density and stiffness may also result in fiber alignment perpendicular to the flow.^140^

The aberrant transendothelial and IFF can lead to excessive accumulation of interstitial fluid and augmentation of interstitial fluid pressure (IFP), what is known as edemas. They are especially important in lungs as they increase nutrients' diffusion distance, in intestine compromising the absorptive function, or in the peritoneum (ascites) promoting infections.^141^

Furthermore, the abnormal transendothelial/IFF and IFP are exacerbated in solid tumor development and wound healing processes.^28^ The rapid and flawed angiogenesis driven by the fast growth of solid tumors (or the wound healing process), leads to leaky BVs, and together with defective LVs unable to drain interstitial fluid and fibrosis, it causes the increase of IFP.^135^ In tumors, IFP varies from 10 to 40 mmHg, in contrast with −2–0 mmHg of normal tissue pressures.^130,135^ Consequently, there is a net convective flow of fluid from the tumor mass into the surrounding tissue, which is recognized as a potential stimulus to promote metastasis by driving tumor cell migration in the direction of flow *via* autologous chemotaxis.^45,142–144^ The augmented transendothelial shear stress generated promotes tumor vasculature remodeling,^145^ and the excessive IFF leads to the mechanical activation of FBs. This increases FBs migration,^146^ their fibrotic features,^28^ and transforming growth factor-β (TGF-β) production,^135^ directly related with immune tolerance, tumor progression, and poor prognosis.^28,45^ Interestingly, IFF also induces M2 macrophage polarization in the tumor surrounding, altering tumor immune microenvironment toward an invasive phenotype.^147^ Furthermore, tumoral IFP and IFF generate aberrant transport of metabolites into the tumor surroundings,^25^ altering nutrients and oxygen arrival and hindering therapeutic drug delivery.^130^

#### 1. 3D *in vitro* models to mimic IFF and transendothelial flow

The simplest strategies employed to model transendothelial and IFF *in vitro* generally involve culturing vascular ECs on a 3D matrix, and/or interstitial cells within the hydrogel. Then, a pressure gradient is applied to drive flow through the gel, for instance, to study the effect of flow on the production of prostaglandins^148^ or on neointima formation.^134^

In tumor studies, 3D *in vitro* models that mimic the *in vivo* tumor architecture and dynamic fluid conditions are important not only to investigate original tumor microenvironment behavior but also to study drug delivery and toxicology of potential new anti-tumor drugs.^149^ Thus, some authors used, for example, gravity-driven flow to study the effect of IFF on tumor cell migration ^136^ and the metastatic capacity of tumorigenic cells^142^ or peristaltic pumps to prolong the time of experimentation and deepen the knowledge on EMT within the tumor microenvironment.^150^ Another approach was the use of syringe pumps to investigate the crucial effect of flow in nanoparticle drug delivery to tumor spheroids as predictive of *in vivo* tumor transport behavior,^151^ with significantly different results in comparison with static conditions.^152^ All these studies highlight the relevance of flow for drug screening assays.

In addition, the biochemical composition and biophysical parameters of culture media and perfusates have an impact on interstitial fluids.^103^ As interstitial fluid usually originates from plasma, the biochemical composition of both fluids is very similar (interstitial fluid contains 40% of plasma protein concentration and has a slightly altered ionic profile).^45^ Interstitial fluid viscosity (∼1.2 cP)^45^ is higher than that of commercial media (∼0.8 cP),^45,106^ and it can affect cancer cell migration, among others effects.^105^ The alternative of using natural media (from tissue extracts) as perfusates in 3D *in vitro* models would increase the mimicking of *in vivo* situation but would reduce the reproducibility of results due to their variable composition.^153^

## III. TYPES OF FLUID FLOW SYSTEMS IN 3D *IN VITRO* MODELS

This section will traverse the most commonly used setups to apply fluid flow in 3D hydrogel-based *in vitro* models. The perfusion systems are classified according to the fluid pressure origin in gravity-based (also known as static head, Sec. III A) or pump-based systems (Sec. III B). In addition, agitation systems are also described (Sec. III C). Their different characteristics determine, among other features, the preferential type of flow generated: luminal, interstitial, transendothelial, or simple external agitation. The systems will be described, providing examples of their use, as well as their limitations, improvements, or alternatives.

### A. Gravity-based perfusion systems/static head systems

Gravity perfusion systems are the simplest and most economical approaches to generate fluid flow in 3D *in vitro* models. These systems usually eliminate the issues associated with operating under a complex setup, which may include pumps, and most often also tubing and connectors (all in conjunction with an incubator).^154^ In addition, these systems allow multiple experiments to be developed in parallel.

#### 1. Fluid columns and Boyden chambers

**FIG. 3.**
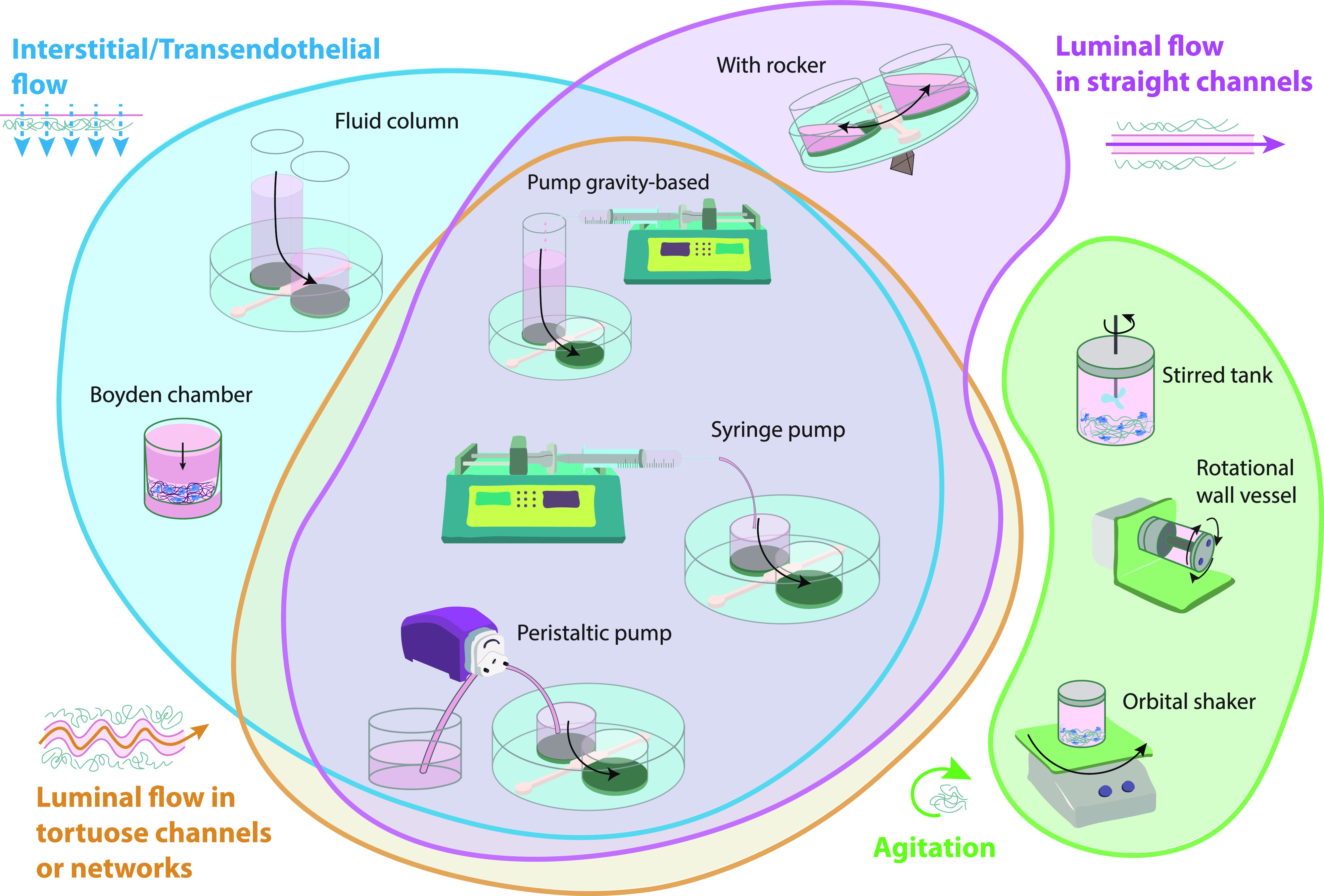
Most commonly used fluid flow setups in 3D in vitro cultures grouped by their preferential type of flow. Transendothelial or IFF (blue) can be achieved by fluid columns and Boyden chambers, with or without associated pump, and pump-based perfusion systems. Straight luminal flow (pink) by rocker-based systems, pump gravity-based systems, and pump-based systems. Tortuous luminal flow (orange) by pump gravity-based systems and pump-based systems. Finally, agitation (green) can be achieved by stirred tanks, RWV, or orbital shaker systems.

Fluid column (or reservoir) systems^65,146,155^ directly connect plastic,^156^ silicone,^65,155^ or glass ^156^ rigid tubes/columns to the commonly used PDMS^157^ devices. The low cell culture medium volume that columns can hold bias these systems against their use to generate luminal flow. On the contrary, the low permeability of the ECM^156^ allows the generation of IFF/transendothelial fluid flow over time, as it occurs in angiogenesis studies and investigations of the formation of functional microvascular network,^65,138,158,159^ wound healing,^156^ or cancer^136,146^ (Fig. 3). However, the flow rates generated are not uniform over time, as the difference of pressure evades progressively. Therefore, column refilling of culture medium is needed to prolong the experimental time,^146,158,160^ or the inclusion of external reservoirs to provide good flow stability to the system (in comparison with basic column systems).^139,161^ Flow stability can be further enhanced *via* a siphon effect and resistor, which are able to regulate the flow rates.^162,163^ To overcome such limitations, the incorporation of external pumps to refill the columns or reservoirs has allowed an automatization of the process. Pumps enable long-term IFF experiments ^164^ and durable luminal flow assays^60,163^ (as the luminal flow generated only by fluid columns can last no more than minutes) **(**Fig. 3).

As an alternative to microfluidic devices, Boyden chambers (or transwells) are used. Due to their design, they are preferentially chosen to mimic IFF in tissues^93,165^ or to simulate transendothelial fluid flow effect^93^ (Fig. 3). Also, they are commonly used in tumor cell migration,^139,142,166^ invasion,^167–171^ and epithelial-to-mesenchymal transition^172^ studies, or to analyze the effect of flow in the tumor microenvironment.^173^

#### 2. Rocker-based perfusion systems

Unlike previously explained methods that produce unidirectional flow, rocker-based perfusion systems provide bidirectional flow. In adult homeostasis, both in LV and BV, unidirectional flow occurs. However, in contrast to blood vasculature, lymphatic homeostatic vasculature is exposed to reverse/oscillatory flow in lymphatic valve areas.^174^ In pathological conditions, such as BV obstruction, a bidirectional flow can be induced.^175,176^ Interestingly, the physiological levels of shear stress generated by bidirectional flow in *in vitro* microfabricated vessels in comparison with static conditions have proven to enhance cellular structure and function.^60^

Rocker-based perfusion systems are predominantly used in cell culture to generate shear stress in straight endothelialized (BVs^177,178^ and LVs^179,180^) or epithelialized lumens^181,182^ (Fig. 3). The formed vessels have served as *in vitro* platforms to study neutrophil^183^ and monocytes extravasation,^67^ a process dependent on the properties of the surrounding ECM,^67,183^ and on the structural integrity of the vessels.^67^ Such endothelial functional integrity was also developed in blood–brain barrier (BBB) studies under rocker-based fluid flow,^184^ showing asymmetric transport of substrates,^185^ and revealing a dual role of astrocyte BBB-regulation after radiation.^186^

Rocker-based perfusion has also been applied to an *in vitro* renal proximal tubule model to study drug-induced kidney injury and drug-transporter interaction,^187^ as well as renal ischemia, being the interrupted flow, together with other parameters, a potent disturbance to the proximal tubule morphology and cell viability.^178^ And as in the mentioned study, other works have shown the integration of BVs with tissues like mammary ducts to reveal their close interaction.^188^ Perfused Caco-2 intestine tubes were proven to work as models for drug discovery and transport across intestinal barriers ^182,189^ or to analyze radical oxygen species (ROS) levels and cell viability.^177^ On another note, angiogenesis models originating from rocker-perfused vessels^177,190^ have been used to irrigate complex biological systems, such as *in vitro* models of respiration^191^ or organoids,^9,192,193^ though these models are more commonly perfused with pump-based systems, due to the long-term maturation times required to maintain their physiology.

Alternatively, microfluidic devices can be designed in a way that the fluid is recirculated *via* a second channel connected to the beginning of the system, as for instance with an endothelialized lumen. Although this is not a continuous flow, this design allows an unidirectional flow within the perfused structure, while avoiding the laborious use of pumps.^194^

### B. Pump-based perfusion systems

Pump-based perfusion systems offer long-term experimentation hardly achieved by any gravity-based system (without pump). Pump-based systems allow long-term IFF,^29,140,195^ and the generation of physiological luminal flow in straight^16,73,163^ tortuous tubular structures,^22,107,113^ or through self-organized^152^ or previously designed^196,197^ microvascular networks. This long-term perfusion experimental time helps to overcome the maturation and development limitation of advanced *in vitro* models,^18,21,197–199^ and therefore, the use of pumps is extended in complex *in vitro* systems, like organoids,^16,21^ organ-on-a-chip,^109^ body-on-a-chip,^200,201^ and advanced tissue engineering.^202^

Mainly, two types of pumps are used in *in vitro* ECM-based perfused studies: peristaltic/roller pumps^203^ and syringe pumps.^204^ Peristaltic pumps can usually move higher volumes than syringe pumps, limited by the volume of the syringes.^205^ On the contrary, syringe pumps provide consistent physiological flow^60^ within a large range of flow rates.^205–207^ Although both peristaltic and syringe pumps can be used to develop IFF^29,140,195,208^ and luminal flow,^18,73,107,113,152,163^ peristaltic/roller pumps may generate continuous flow oscillations or waves (also called pulsations)^209^ capable of producing a more random organization or unalignment of ECs.^210^

The undesired oscillatory flow, also generated at initial stages of syringe pump flowing,^211,212^ must be resisted by the system, and it can be dampened to obtain fully steady laminar flow^210^ by including reservoirs^213^ or long compliant silicone tubing.^21,212^ Interestingly, peristaltic-pumps' pulse can be used to model pulsatile blood flow in the arterial vasculature^214^ or to produce rhythmically aligned stretching and contraction of intestine cells to develop a more physiological peristaltic gut.^16^ The number of rollers^16,215^ and the rotation speed^209^ may introduce flow variabilities that require the characterization of each pulsatile flow.^216^

Additionally, other alternatives like pressure-controlled systems/pumps or vacuum pumps have been explored to generate fluid flow in 3D cell cultures^217–220^ or other perfused *in vitro* microphysiological systems.^221–223^ Essentially, pressure-controlled systems are formed by an external pressure source (usually controlled by software) and a pressurized reservoir that contains the perfusate. Advantageous to flow-controlled systems, they can exhibit a fast settling of pulseless flow. Also different from flow-controlled systems, pressure-controlled systems must adjust their pressure drop to maintain advection levels when working with different viscous perfusates or different tissue resistances (see Glossary Box).

In comparison to gravity-based perfusion systems, the use of pumps as pressure generators in *in vitro* models imply an increasing complexity of the system to be set up in number of components, connectivity, and manipulation. This complexity favors potential mechanical strain and detachment of the hydrogel, the generation of bubbles in the system^60^ (a major challenge^224^ routinely encountered in microfluidics^225^), and thus, a substantial reduction of the experimental throughput. To overcome some of these issues, fittings and connectors should be reduced and placed downstream of a (preferably) hydrophilic-made device, designed with rounded and obtuse angles.^224^ On another note, although infuse pumps generate a more physiological difference of pressure between perfused vessels and its surrounding microenvironment than withdrawing pumps,^135^ they can aggravate the differences of pressure and temperature within the device, which promote bubble formation,^224^ and, thus, they may require the use of bubble traps.^226–228^ Conversely, withdrawing pumps enable the reduction of connectors' use upstream of the device, while preventing pressure decrease within the device. These advantages of withdrawing pumps reduce air trapping and the gaseous saturation of the medium, which are key elements to avoid bubble formation.^224^ Furthermore, this setup requires upstream medium reservoirs that can be placed within the incubator, providing the necessary thermal stability to prevent gas solubility reduction. The use of two pumps, with infusing and withdrawing modes, respectively, requires a very precise flow coordination,^29^ and it induces a higher risk of hydrogel destabilization when plugging the tubbing to assemble the device into the system.^60^

Moreover, to standardize perfused *in vitro* cell cultures, and, therefore, reduce lab variability, the recently generated fluidic circuit board (FCB) of the translational organ-on-a-chip platform provides an interesting and well characterized tool to develop any kind of perfusion system in 3D *in vitro* models.^229–231^ This multi-institutional proposal integrates commercially available components that facilitate connections and reduces the above explained pump-related bubble issues,^229^ while increasing the throughput of perfusion platforms.^231^ Additionally, complex liquid-handling instruments that contain pumps are being developed to automate the perfusion process and reduce direct fluidic plumbing and bubble formation troublesome.^232^ Their use in a multi-organ-chip helped to predict clinical quantitative pharmacokinetic parameters, closing the gap between *in vitro* and *in vivo* experimentation.^233^

Table I groups the types of perfusion systems used to induce flow in 3D (ECM-based) *in vitro* cell cultures and summarizes the advantages, disadvantages, and examples of the different setup systems.

**TABLE I.** Types of perfusion systems to induce flow in *in vitro* cell cultures. This table groups the different types of perfusion platforms highlighting their advantages, limitations, and potential improvements as well as recommendations and examples of their use.

Perfusion System	Advantages	Disadvantages	Improvements	When to use (preferentially)	Quantitative data	Examples
Columns (or reservoirs) 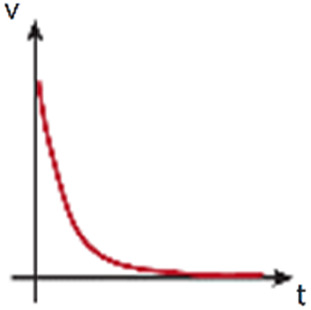	High throughput - Simple handling - No need of bubble trap	- Limited in time and speed - Flow decreases over time - Low microscopy visualization in Boyden chambers	- Repetition over time (although affecting the flow profile) - Including external reservoirs - Including flow resistors (affecting shear stress and advection)	- Short experiments of interstitial or transendothelial fluid flow preferentially	- Initial pressure drop: ∼1–25 mm H_2_O - Manual pressure drop renewal for IFF: ∼4–24 h - Flow rate: ∼2–15 *μ*l/min - IFF speed: ∼0.1–5 *μ*m/s Parameters highly dependent on gel permeability, gel dimensions, and reservoir size	Refs. 65, 136, 138, 139, 146, 155, 156, 158–162, and 164Refs. 93, 142, 165, and 167–173
Boyden chamber 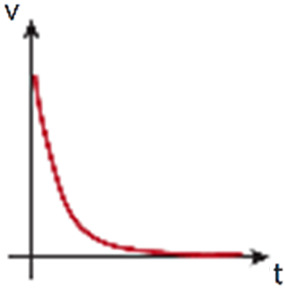						
Gravity-based with pump 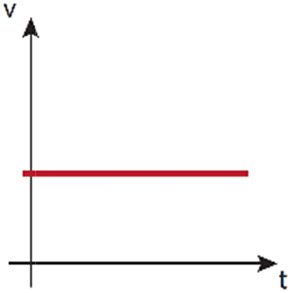	- Simple handling - Unlimited/long working time - No bubbles formation - Nutrients renewal	- Low throughput - Limited in speed - Low sterility	- Including air filters to control sterility	- Very long experiments with renewal of nutrients	- Shear stress: ∼0–2 dyne/cm^2^ - Nonstop working time: weeks Pressure drop and flow rates are similar to the rest of gravity-based systems.	Refs. 60, 163, 164, and 166
Rocker-based 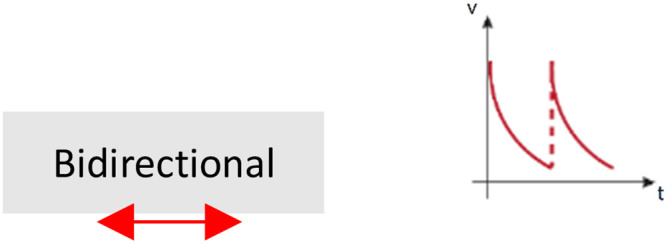	- High throughput - Simple handing - No need of bubble trap	- No renewal of nutrients - Low sterility	- Bypassing design to recirculate flow and perfuse unidirectionally^a^	- Shear stress cell stimulation without renewal of nutrients	- Angle: ∼±7°to ±37° - Tilt interval: ∼12 s–8 min - Shear stress: ∼0 to 6 dyne/cm^2^	Refs. 60, 67, and 177–194
Pump-based via peristaltic pump ^b^ 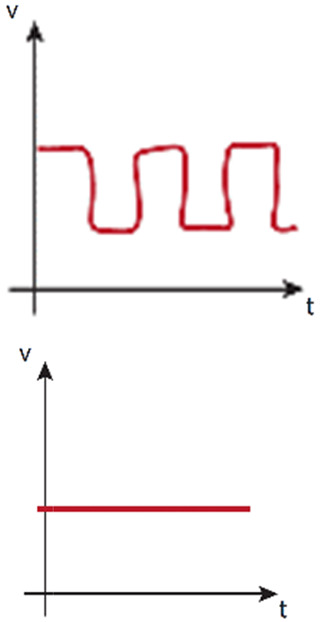	- Unlimited working time - Nutrients renewal - Allowed bidirectional flow (although compromising medium renovation)	- Compromised hydrogel stability when handling - Low throughput - Potential bubble formation	- Inclusion of bubble traps - Precise design and position of system elements (device, pumps, fittings) - Pulsation dampeners	- Very long experiments with renewal of nutrients - Peristaltic stimulation	- Flow rate: ∼0.5–4000 *μ*l/min - Shear stress: ∼ 0 to 20 dyne/cm^2^ - Nonstop working time: weeks Parameters highly dependent on the pump, tubing, and microphysiological system	Refs. 16, 21, 22, 73, 107, 113, 140, 150, 196, 197, 208, 213, and 234
Pump-based via syringe pump 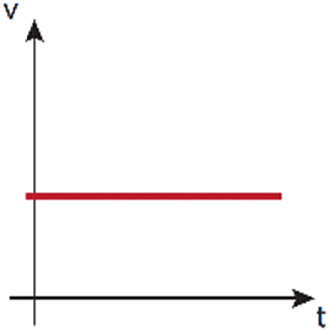	- Long working time - Nutrients renewal - Allowed bidirectional flow (although compromising medium renovation)	- Compromised hydrogel stability when handling - Low throughput - Potential bubble formation	- Inclusion of bubble traps - Precise design and position of system elements (device, pumps, fittings)	- Long experiments with renewal of nutrients	- Flow rate: ∼1–50 *μ*l/min - Shear stress: ∼ 0 to 2 dyne/cm^2^ - Nonstop working time: days Parameters highly dependent on the pump, syringe, and microphysiological system	Refs. 29, 152, 163, 195, 225, and 235

^a^ Depending on the aim of the experiment, it may just be considered as an alternative.

^b^ Depending on the number or rollers and the presence of pulsation dampeners, fluid flow could be either laminar or pulsatile-like.

### C. Stirred tank, orbital shaker, or RWV-based agitation systems

In a different scale, there are extra alternatives used to generate fluid flow, as stirred tanks and orbital shakers,^236,237^ which do not provide perfusion but provide agitation instead. They are generally used to overcome the low nutrient diffusion encountered in tissue engineering^238,239^ and development of organoids.^240,241^ Thanks to these systems, fluid flows around the organoid or tissue, facilitating the mass-transfer and exchange of nutrients and waste products with the medium.^236^ Standard Petri dishes or well plates can also be used as orbital shaking platforms,^242^ or they can be adapted to be used as stirred tanks for 3D cell culture of organoids.^243^ Stirred tank or orbital shaker agitation systems are used to help in the maturation of ectoderm tissues like brain organoids^240,241,244^ and endoderm tissues like liver organoids.^245^ Nevertheless, except for renal derived organoids^246^ agitation systems are not commonly applied to mesoderm derived tissues.^198,247^

On a special note are rotating wall vessel (RWV, or clinostat), bioreactors that provide high mass-transfer while simulating microgravity and enhance cell-cell interaction^37^ (Fig. 3). RWV showed improved growth and differentiation of retinal organoids in comparison with static culture, recapitulating the spatiotemporal development of the retina *in vivo.*^248^ Similarly, cardiac tissue culturing in RWV, compared to static culture, significantly enhanced cardiomyocytes maturation, functionality, and viability.^249^ Furthermore, these bioreactors reduce the mechanical shear forces and bubbles that can be generated in stirred tanks that can induce cellular damage.^250^

## IV. FLUID FLOW APPLIED TO CURRENT BIOLOGICAL MODELS AND ITS CHALLENGES

In the last decades, bioengineering *in vitro* 3D models have experimented an unprecedented growth, being nowadays an expanding field with multiple clinical applications. Many of these newest technological models already include perfusable systems, while more sophisticated models, such as those with microvasculature, are being developed. In this section, we discuss important studies that have made use of the newest bioengineering applications such as organ-on-a-chip, organoids, and body-on-a-chip and their importance in drug discovery and in personalized and regenerative medicine.

### A. Perfusable bioengineered tissues

The so called “mini-tissues,” also named as organ-on-a-chip (OoC) have been engineered in microfluid devices to reproduce *in vitro* key aspects of organ development, functionality, or disease.

Since the innovative work of Huh D and colleagues in 2010 who developed a lung-on-a-chip model,^251^ many other organs, e.g., intestine and kidneys, have been modeled *in vitro*.^252,253^ The drawback of OoC models is their simplicity compared to native organs, since they are made almost exclusively on guided microchannels where one or several cell types are cultured in ECM coated membranes to model a particular tissue.^251–253^ This diminishes the complexity, and, thus, the physiological relevance of OoC models compared to organoids derived from murine or patient stem cells, as explained in Sec. IV B. However, OoC allows understanding cell-to-cell interactions between different cell types in the mechanical/biochemical conditions of the tissue of study, providing useful information on how cells communicate to each other in different conditions.

Different microfabrication techniques are being used to form mini-tissues in pre-established device structures, such as soft and photo lithography,^254^ injection molding,^255^ micromilling,^256^ laser photoablation,^257^ or 3D bioprinting.^258^ When fluid flow is a requisite in the study, as it is the case for instance to simulate liver sinusoid structures, renal reabsorption, or alveolar-capillary functioning, the incorporation of flow in the system has to be considered during the design of the microfabricated mini tissues/OoCs. Fluid flow, besides its crucial role in transporting nutrients and removing waste metabolites, can provide mechanical cues (laminar stress, transmural) that enhance cell viability and intercellular communication and enable dynamic control of the environment by allowing controlled incorporation of exogenous compounds, e.g., drugs or toxins, in 3D OoC models.^80,259^ In order to apply fluid flow to OoC models, continuous perfusion to the microchamber is required, and, therefore, to set up such a model is laborious and requires expertise and proper equipment. The advantage of such platform relies on the straightforward readouts and on the control of the tissue microenvironments that allows recapitulating the complex biochemical interactions between different cell types and the inclusion of microenvironmental physical forces, such as shear stress and mechanical traction or compression.^260^

**FIG. 4.**
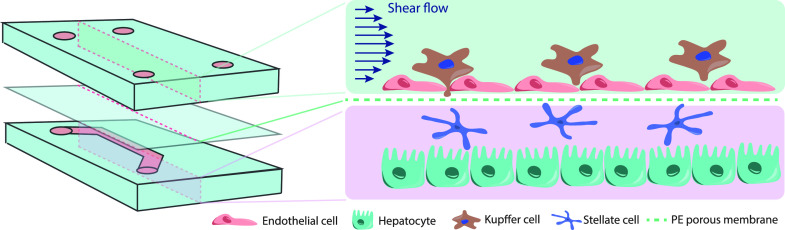
Liver sinusoid-on-a chip. The model is formed by the four types of hepatic cells, i.e., liver sinusoidal endothelial cells, Kupffer cells, hepatic stellate cells, and hepatocytes, distributed layer-by-layer resembling liver sinusoid. Stellate cells are first injected into the basolateral side of the collagen-I pre-coated PE membrane. Sinusoidal endothelial cells are then added on the apical side of the membrane together with Kupffer cells. Hepatocytes are then added to form the bottom layer. Unidirectional flow is incorporated by a syringe pump. Based on Du *et al.*^118^

Du *et al.* engineered a liver-on-a-chip to mimic the interactions between liver sinusoids and blood flow peripheral cells. Their model resembles the liver sinusoid structure and functionality by integrating the four cell types implicated (sinusoidal endothelial cells, Kupffer cells, hepatic stellate cells, and hepatocytes), the fenestrated morphology characteristic of liver sinusoids (by using a porous permeable membrane), and shear flow applied to mimic the capillary blood flow pattern in the liver sinusoid^118^ (Fig. 4). Capillary blood flow in the liver sinusoid is necessary for mass transfer and for nutrient supply and especially for IFF inside the Disse space deriving from capillary flow across the permeable endothelium. However, the model uses murine cells, and it is of use only when short-term analyses are needed, which is valid for innate immune response studies, cytotoxic analysis, or protein secretion.^118^

Huh *et al.* engineered a pioneering model to reproduce lung alveolar-capillary functionality using two microchannels separated by a porous ECM-coated membrane, one channel lined with alveolar epithelial cells and air filled and other channel filled with fluid and lined with lung endothelial cells. Moreover, cyclic mechanical suction was applied to both channels to simulate lung breathing.^251^ Importantly, the device resembles the architecture, mechanical environment, and functionality of alveolar-capillary unit allowing functionality analysis, e.g., nanoparticles absorption, and pathological studies, e.g., pathogen and immune cell interaction. Nevertheless, the absence of alveolar macrophages (crucial to fight against incoming pathogens and pollutants), the air pressure, and flow values applied, the use of transformed lung endothelial cells, or the thickness of the barrier differ significantly with the *in vivo* situation and, thus, do not completely mimic lung physiology.^251^ More recently, a perfusable vascularized epithelial lung high-throughput 64-chip microfluidic plate-based platform was developed. The platform was made with human primary microvascular ECs, fibroblasts, and pericytes with the purpose of studying viral infectivity and viral infection-induced thrombotic events^191^ in large-scale patient collection of samples in cases as COVID-19 pandemic.

To mimic cardiac physiology, proper propagation and maintenance of mechanical and electrical signaling is fundamental. Several heart-on-a-chip models have been developed providing key signals needed to reproduce mechanical (through cyclic pressure stimulation)^261^ and electrical (through controlled uniform electric field)^262^ cues in the heart. However, these systems fail to properly sustain the demand of oxygen and nutrients of such highly metabolically active cells for long periods of time (longer than few days in culture). To overcome this issue, an automated gravity-based controlled flow was implemented in a model that was able to improve the functionality of cardiomyocytes.^225^

On the other hand, Lin *et al.* investigated renal reabsorption in a kidney-on-a-chip model where adjacent conduits of epithelial and endothelial cells ECM-embedded presented active reabsorption of solutes (tubular-vascular exchange) in a closed-loop perfusion system.^113^ The system allows solutes and drugs to be flown and perfusates to be collected at different time points. Also, a high-throughput, 3D-microfluidic platform (Nephroscreen) for the detection of drug-induced nephrotoxicity was generated.^143,187^ In this platform, FSS was incorporated through passive leveling by gravity. Though immortalized renal cell lines were used, renal cells were able to show a polarized epithelium with functional transporters.^187^

**FIG. 5.**
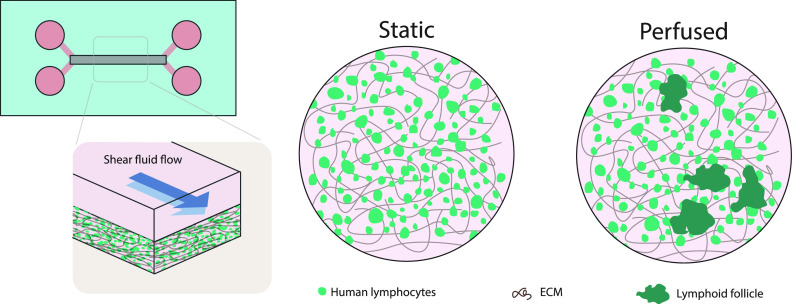
Lymph node on-a-chip. An ECM that contains human lymphocytes is stimulated with medium flowed in the upper channel. Differences are observed in the growth of human lymphocytes (in green) between static and flow conditions. Under flow, lymphocytes are grouped, simulating formation of follicles. ECM fibers are shown in brown. Based on Goyal *et al.*^265^

Despite the crucial role of immune cells in shaping tissue homeostasis and their crucial role in disease fighting,^263,264^ the incorporation of immune components into OoC models is generally unexploited. Recently, an elegant study showed the *in vitro* formation of lymphoid follicles when culturing B and T lymphocytes in a 3D hydrogel microchip under fluid flow.^265^ Flow was needed for follicle formation and for preventing autoactivation of lymphocytes. Due to the formation of follicles and the feasible incorporation of dendritic cells to the model, the platform can be useful for preclinical testing of vaccines, adjuvants, or immunotherapy drugs ^265^ (Fig. 5).

Recently, patient derived-induced pluripotent stem cells (iPSC) have been differentiated to be cultured in OoC systems to study the response of patient derived cells to different drugs.^266^ Together, all the above-mentioned works highlight the potential use of OoC models as platforms for high-throughput personalized drug screening.

Though in the majority of studies using organ-on-a-chip the choice of perfusate has been commercial cell culture medium, some models during the last decade that contain endothelialized hollow channels have been exposed to flowing blood to study thrombosis *in vitro.*^267–269^ While these devices are useful for advancing research, they are not used in practical or clinical settings because living cell cultures are not robust and the devices cannot be stored for extended times. For instance, Jain *et al.*^53^ studied vascular thrombosis in an endothelialized microfluidic device using whole blood taken from subjects receiving antiplatelet medications. However, only short-time analyses have been performed. To the best of our knowledge, the lymph has not been used in OoC and the inmune and fatty content of lymph (chyle, formed in the intestine) could damage vessels rapidly, if not used at the right concentration and flow. Both perfusates, blood and lymph, are challenging to work with because of their viscosity and complex rheology due to suspended particulates, which results in high shear stresses even in large vessels. To replicate their properties *in vitro* with cell culture medium, faster flows or increased spatial velocity variability is necessary to maintain the same flow resistance. Especially important and complicated is the selection of perfusate when generating a body-on-a-chip (discussed in Sec. IV C), since the perfusate varies across tissues. Therefore, to simulate faithfully this difference, tissue-specific perfusates with different biochemical and biophysical properties should be used.

### B. Perfusable bioengineered organoids

The *in vitro* differentiation of stem cells to form organoids has enabled researchers to faithfully reproduce in 3D *in vitro* key structural and functional properties of many organs.^270^ Since the very first gastric organoids developed by Sato *et al.*,^14^ other complex organoids such as brain,^240^ liver,^271^ stomach,^16^ pancreas,^12^ or lung organoids^13^ have been generated. Many promising advances have been done using these multi-cellular engineered living systems that allow mimicking organ biology in a Petri dish.^272–274^

The cellular organization, maturity, and function of organoids has been improved with the implementation of physico-chemical stimuli (e.g., tissue strain/compression or shear stress). In several cases, the inclusion of fluid forces in organoid models has been proven fundamental for their growth, functionality, and long-term maintenance.^16,21^ Interestingly, the generation of human pancreatic islet organoids in a perfused 3D system exhibited proper growth, differentiation, and maturation compared to static culture, being this platform key for diabetes studies and drug testing.^15^ However, care should be taken on the flow conditions and incubation time applied to the different tissue of origin organoids since, for example, the extended culture of iPSC-derived kidney organoids in stirred suspension has been proven suboptimal.^246^

**FIG. 6.**
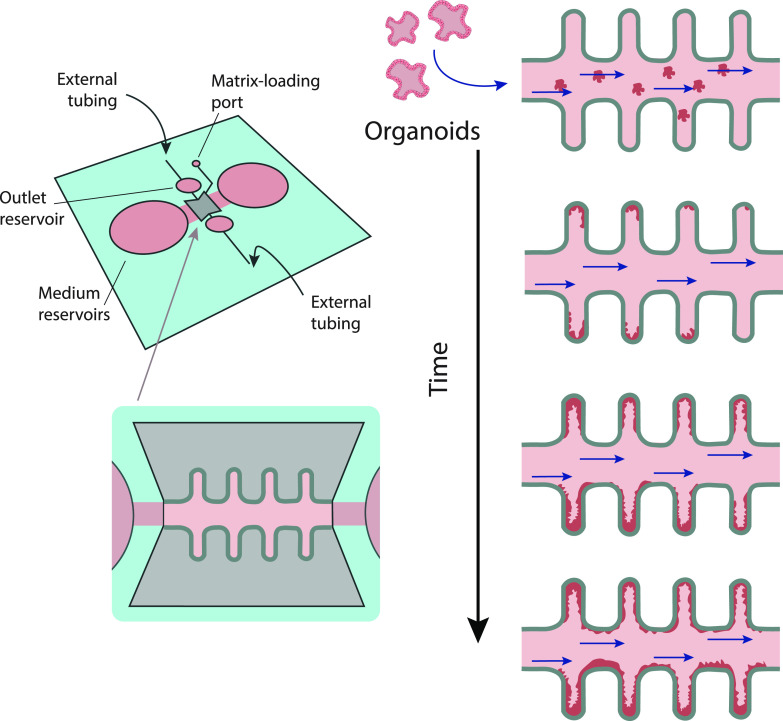
Engineered mini-guts. Microfluidic device with inlet and outlet reservoirs designed to allow intestinal lumen perfusion. Cells were allowed to stay in the cavities and cell seeding medium was changed every day for providing a viable long-term platform (20 days). Based on Nikolaev *et al.*^116^

Different from OoC models, organoids-on-a-chip follow intrinsic programs to develop an organ. While an organoid develops, several signaling pathways are activated timely to generate the different cell types of a tissue.^275^ In this context, biochemical gradients are to be considered for cell type morphogenic differentiation and niche maintenance. For instance, a platform has been designed for neuronal tube development that integrates opposing gradients of bone morphogenic protein (BMP) and Hedgehog signaling molecules to properly form neuronal tube patterning.^276^ Also, in a gut-on-a-chip model, opposing gradients of WNT and BMP have been achieved mimicking villi and crypt,^277^ revealing the useful application of scaffold-guided morphogenesis. An excellent example of a spatially arranged organoid-on-a-chip model are the mini-guts generated by Nikolaev and colleagues.^116^ This model consists of a permeable scaffold that enables the adhesion, growing, and differentiation of intestinal stem cells, and, at the same time, it serves as physical barrier that guides the self-organization of stem cells into a functional epithelium with absorptive and secretory functions. The scaffold is laser-ablated to mimic villus-like and crypt-like region geometry, closely resembling the intestinal epithelium. The mini-guts generated harbor a lumen connected to an external pump for their perfusion, and thanks to the incorporation of flow and removal of dead cells, the mini-guts are able to be fully functional for several weeks and capable of regeneration without the need of organoid passaging^116^ (Fig. 6).

Several organoid models are implementing a different approach, which involves incorporating functional vasculature in the system. Thus, to study brain development and disease, EC reprogramming was induced inside human cortical organoids.^18^ When functional vasculature-like structures were formed, enhanced maturation of organoids was obtained. This model offers an interesting approach to study the crosstalk of neural and ECs, and a way to reduce hypoxia and apoptosis usually found in avascular organoids.^18^ Another approach to induce vasculature within cerebral organoids was by adding VEGF (to induce vessel formation) and Wnt7 (to induce development of pericytes) during the generation of cerebral human stem cells. This model allows the formation of BV-like structures with mature blood–brain barrier characteristics in cerebral organoids. The method permitted organoids maintenance up to 4 months in culture;^19^ however, the development of BVs in cerebral organoids was constrained due to a reduction in the number of vascular-like structures in extended cultures. In addition, the open-circle morphology of endothelial layers in 4-month organoids appeared distorted, potentially resulting from the absence of blood pressure. Consequently, additional investigation is necessary to facilitate the generation of fully functional cerebral organoids with a vascular system capable of supporting the flow of medium.

Besides the relevance of the biochemical composition of the microenvironment, the biomechanical properties of the ECM, e.g., stiffness, permeability, viscoelasticity, porosity, and topography have an important impact on cell behavior; ^278^ thus, they are crucial to reproduce the *in vivo* organ functions. For organoid formation, the most commonly used ECM hydrogel for physical support of cells and biochemical signaling is Matrigel.^279^ However, due to batch-to-batch variability ^280^ and lack of knowledge about its entire composition, standardized synthetic and natural hydrogels are being designed according to the tissue properties to be reproduced *in vitro*.

**FIG. 7.**
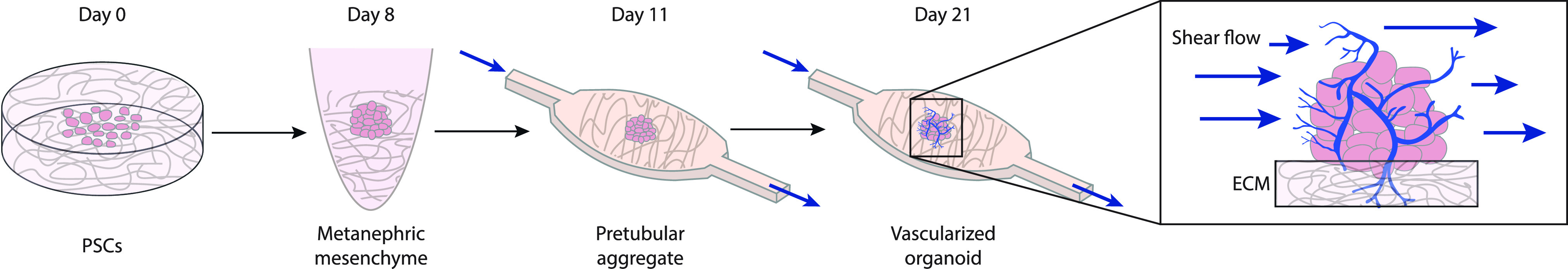
Renal organoids cultured under high fluid flow exhibit robust vascularization and maturation. Renal vascularized organoids are placed on an ECM and subjected to controlled fluidic shear stress. Peripheral vascular network is formed after 21 days under flow and ECM-adherent conditions. Based on Homan *et al.*^21^

External forces have a crucial effect on organoids formation since the cells can mechanosense these forces by focal adhesion proteins and ion channels and react, activating different mechanotransduction pathways, such as Ras/MAPK, P13K/Akt, RhoA/ROCK, Wnt/β-Catenin, and TGF-β, which are essential for tissue morphogenesis and maintenance.^281,282^ For example, several organoid-on-a-chip based studies have implemented organ-specific fluid mechanical forces. Thus, Lee K. and colleagues perfused gastric organoids *via* a peristaltic pump, which allowed organoid stretching and contraction mimicking gastric movement.^16^ Recently, liver organoids derived from human pluripotent stem cells have been generated in a 3D perfusable micropillar chip model.^283^ In this system, controllable fluid flow allowed proper growth and differentiation of liver organoids, recapitulating liver formation and cellular heterogeneity; it also enhanced not only cell viability but the expression of key genes for hepatocyte differentiation and metabolism, thus highlighting the relevance of mechanical flow in promoting proper organoid functioning.^283^ Similarly, Homan *et al.*^21^ demonstrated that when partially ECM-embedded kidney organoids derived from human pluripotent stem cells, are perfused, an increased vascularization and maturity of its tubular and glomerular compartments is obtained (Fig. 7). Their proper development was dependent on the flow-induced shear stress generated, revealing that organoids cultured under flow were more mature and polarized than organoids grown in static culture conditions. Thus, this study exemplifies how 3D vascularized organoid models best resemble the structure and function of specific organs.

Ensuring the establishment of vascular networks is crucial for the appropriate engraftment and function of transplanted tissues. Remarkably, Takahashi *et al.* utilized a dynamic self-condensation method to create tissue organoids from dissociated organ progenitor cells with the presence of vascular and mesenchymal progenitors. This approach facilitated the prompt development of BVs in tissue organoids in the absence of flow.^284^ To achieve this goal in the pancreas, Takahashi *et al.* used the above-mentioned approach, co-culturing pancreatic islets with HUVECs and mesenchymal stem cells. When those vascularized islets were co-transplanted in a mouse model of diabetes, mice showed improved pancreatic viability and functionality (insulin secretion capacity). Taken together, this success sheds light on the use of self-condensing cultures for therapeutic transplantation purposes without the requirement of flow in the system.

### C. Perfusable bioengineered body-on-a-chip

**FIG. 8.**
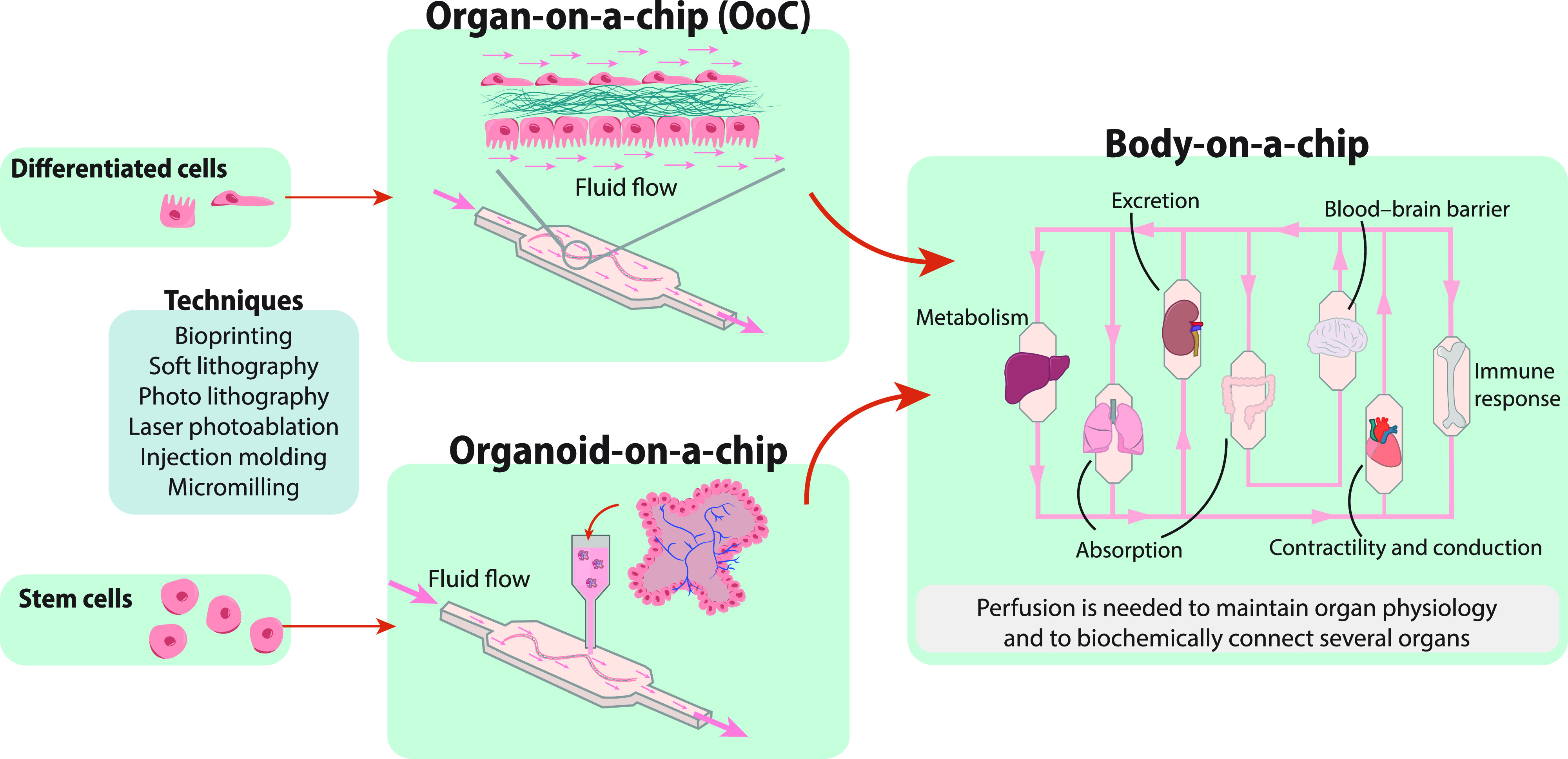
Bioengineered 3D systems to model *in vivo* organ and body homeostasis or disease. Organ-on-a-chip (OoC) made of differentiated cell types embedded in an ECM are able to mimic some physiological functions of organs. Organoid-on-a-chip is used to model the intrinsic program of an organ. Different techniques are used to make both OoC and organoid-on-a-chip, such as bioprinting, laser ablation, and others. Organ-specific derived organoids or OoC can be connected by flow to establish a body-on-a-chip.

With the aim of simulating multiorgan interactions and physiological responses at the systemic level, multiple OoCs/organoids-on-a-chip can be connected by fluid flow to construct a body-on-a-chip (as shown in Fig. 8).^12,285^ To link two or more tissue compartments *via* a common vasculature, the so called “AngioTube” has been developed. The AngioTube features a central microchannel with sufficient mechanical stability to support a perfusable vascular system and enable the self-assembly of diverse parenchymal tissues.^193^ Moreover, a platform consisting of compartmentalized microfluidic chips of different cell types connected *via* fluid flow through micro-engineered porous barriers was engineered to simulate paracrine exchange between cells.^286^

A very interesting 3D-multi-tissue study shed light on the importance of generating body-on-chip platforms for drug screening. The study consisted of multiple microchambers containing different microtissues, interconnected by microchannels and cultured under continuous gravity-driven flow. When culturing liver and colorectal tumor tissues with a chemotherapy agent, the drug impacted tumor growth only after its bio-activation by the liver.^287^ Another important study was performed by Zhang and colleagues, they generated a perfused body-on-a-chip model using interconnected hepatic and cardiac organoids and a peristaltic pump. They designed an automated multisensory platform for long-term real time monitoring of biophysical and biochemical parameters (oxygen, temperature, and pH).^200^ However, there are some limitations of the prototype platform such as the use of PDMS, which has been demonstrated to absorb hydrophobic molecules; therefore, the prototype is not valid for acurate biochemical studies. On the other hand, the complex manual assembly of the prototype system is another limitation. Further improvements of the platform would allow for more accurate modeling and analysis of tissues functioning.

All these studies highlight the importance of designing multi-organ platforms for studying the physiology and pathology of organs and for drug screening studies.

### D. Perfusable models: key for personalized medicine and drug discovery

Even though personalized cell cultures, as human organ-on-a-chip systems, have not yet been used to take decisions about personalized treatments and precision medicine, their potential to accelerate drug discovery is evident.^187,189,288,289^ Indeed, such accomplishment is seen close in time^288,290^ since they have been recently approved by the Food and Drug Administration (FDA) to substitute animal experimentation in pre-clinical assays.

Here, we describe some examples of the potential use of perfused 3D models for patient treatment and drug discovery with a final focus on tumor-on-a-chip technology.

A perfusable 3D kidney-on-chip model using renal organoids^108^ was developed to understand the impact of flow on drug transport and uptake in kidney organoids. The model mimics the native uptake of nephrotoxic substances in human kidneys and, thus, is a vital stride toward determining their potential for drug screening and disease modeling. The model provides a more precise predictor of nephrotoxicity compared to conventional models, which are based on immortalized proximal tubule epithelial cells.

Fat-on-a-chip models with integrated flow are able to reproduce obesity and its related diseases, e.g., diabetes, osteoarthritis, or fatty liver.^291,292^ Adipose cells *in vivo* are protected from shear stresses by the vasculature, which shields the adipose compartment from the bulk flow of fluids. A shortcoming of exerting flow over adipocytes using microfluidic devices is the damaging effect of shear forces.^291^ Therefore, *in vitro* approaches should consider the use of vascularized adipocytes instead of directly applying flow. To simulate this *in vitro*, Loskill *et al.* created an endothelial-like barrier that connected the media channels and adipose chambers using micropores, allowing them to maintain functional lipid metabolism for a period of weeks.^293^ Static cultures have the advantage of lower risk of shear stress-induced adipocytes apoptosis, but would need to address the physiological components associated with nutrient delivery through an active and selective vascular barrier to control nutrients transport to the adipose compartment.

On another note, to study infections, such as cerebral malaria, Bernabeu and colleagues generated a new flow-based 3D brain microvessel model. After 3 days in culture, primary human brain microvascular ECs formed fully endothelialized lumens that could be perfused with blood components, such as *P. falciparum*, to analyze parasite sequestration using a diverse range of flow velocities and wall shear stresses.^294^ This model can be used to study host cell or pathogen interactions with brain endothelium. Moreover, future 3D microvessel engineering approaches toward a more physiological model could incorporate patient-derived perivascular cells like pericytes and astrocytes. Also, the analysis of other blood components would be interesting to study their role in infection.

**FIG. 9.**
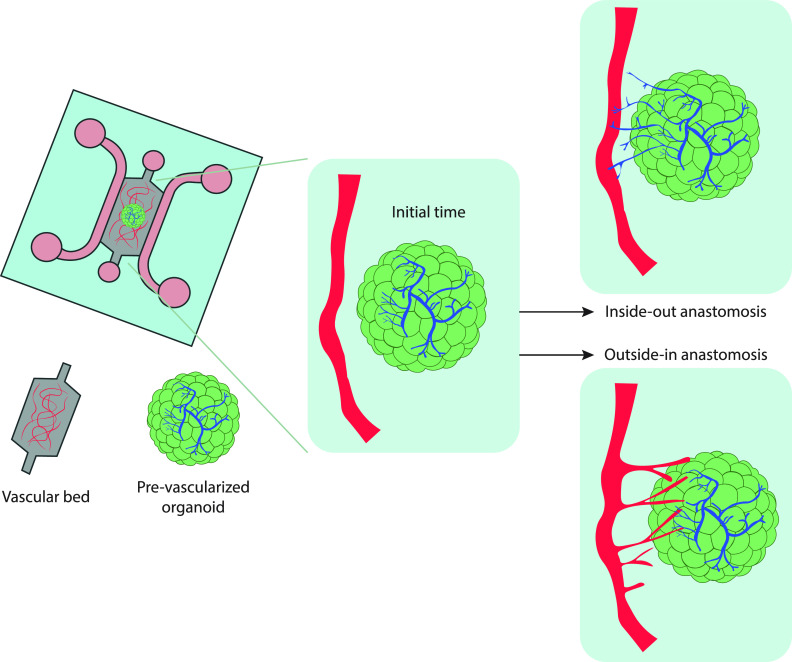
Tumor-on-a chip model. The schematic representation illustrates possible mechanisms for establishing anastomoses (functional connections to form between the vessels) between an *in vitro* vascular bed and pre-vascularized organoids when co-cultured in a microfluidic chip. In the “inside-out” approach, the organoid-derived vessels extend and merge with the existing vascular bed. The “outside-in” approach relies on the induction of angiogenic sprouts from endothelial cells (ECs) of the vascular bed that penetrate the organoids and establish connections with the organoid-derived vessels. This model allows for the exchange of nutrients, oxygen, and other factors between the vascular bed and organoids. Based on Zhang *et al.*^309^

Tumor-on-a-chip technology has become a robust *in vitro* model for cancer research.^295,296^ Despite the complexity of mimicking the biochemical and mechanical characteristics of a tumor *in vitro*, (dense ECM of tumors, immunosuppressive environment given by stromal and immune cells,^297–299^ hypoxia,^300^ and abnormal vasculature^301^) tumor-on-chip platforms closely recreating *in vivo* tumors have been generated.^295,302,303^ The first assays using tumor-on-a-chip systems were developed in static conditions, thus ignoring the effects of blood and lymph flow to tumors. However, the most recent models reproduce reliably tumor blood vasculature by integrating pre-vascularized organoids with a capillary bed to achieve functional anastomoses (Fig. 9). When co-culturing organoids and a capillary bed, the type of hydrogel and the cultured media (a mixed of endothelial and organoid growth media) needs to be optimized. Usually, a hydrogel mixture of fibrin and Matrigel allows for the growth of both ECs and organoids, as Rajasekar *et al.* showed with patient-derived colon organoids.^304^ The device utilized in the study was equipped with a programmable rocker, which facilitated cyclic luminal flow across the capillary bed that improved growth and functionality of the organoids. While the presence of perfusable vessels near the organoids indicates the potential for establishing connections between the organoid-derived vasculature and the adjacent vasculature, direct evidence of intravascular perfusion within the organoid was lacking. Further investigation and techniques are needed to confirm whether there is functional perfusion within the organoid and if there is direct integration between the organoid vasculature and the surrounding vessels. The same approach could be used with LVs so that both circulatory systems are reproduced with the tumor organoids. Moreover, some tumor-on-a-chip studies have simulated *in vivo* dynamic flow,^101,305^ drug efficacy,^152,306,307^ and tumoral vascular immunosuppressive characteristics.^308^ Recently, a unique tumor-on-a-chip with perfusable bioprinted blood and LV pair was designed to mimic the transport of drugs in the TME.^99^ This system is a promising tool to test therapeutic approaches using cancer-derived organoids from biopsies, which could contribute toward personalized medicine treatments.

Developing preclinical models using patient biopsies, along with patient tissue sequencing, serves as a fundamental guide for personalized medicine.^310^ In the study of Pauli *et al.*, patient-derived tumor organoids are being utilized, in conjunction with genomic exome sequencing, to guide precision therapies.^311^ The study shows great promise in guiding the choice of clinical trials for individual patients.^311^ Once these type of high-throughput studies are validated, they could be complemented with selected types of human tumor organoids cultured in microfluidic platforms to find the optimal therapeutic strategy to follow.

During metastasis, tumor cells are exposed to mechanical forces such as fluid pressure from their tissue microenvironment. In order to study the role of fluid flow in the metastatic capacity of tumor cells, metastatic processes such as tumor extravasation have been mimicked in *in vitro* 3D models.^312,313^ A significant study evaluated in a human microvascular network model the effect of luminal flow induced-shear stress, trans-endothelial flow, and IFF on tumor cell extravasation and migration. The results show that luminal flow enhanced tumor cell extravasation, while transendothelial flow augmented tumor cell transmigration across the endothelium. When subjected to physiological flow, tumor cells were shown to migrate closer to the endothelium, favoring the formation of metastatic foci.^220^ These data underscore the significance of utilizing *in vitro* models that can mimic human pathophysiological conditions with high spatio-temporal resolution, as well as applying physiological luminal flow, when investigating extravasation.

### E. The importance of flow for tissue engineering and regenerative medicine

The presence of a physiological environment that includes mechanical forces induced by fluid flow and pressure is important for tissue regeneration, morphogenesis, and repair.^80,259^ This has been demonstrated in various studies, including the mini-guts generated by Nikolaev *et al.*,^116^ where luminal exposure to flow helped to regenerate the epithelium. Another study showed that human mesenchymal progenitor cells can differentiate into osteoblasts in a 3D environment with FSS, which makes it a promising model for bone engineering.^314^

Generally, tissue engineered models harbor poor survival because of their limited mass transfer of oxygen and nutrients. As a consequence, big devices, often called “bioreactors,” were designed to overcome this problem. Bioreactors are crucial for tissue engineering as they allow for the growth of large-scale tissues and organs.^315,316^ By providing an environment that mimics the *in vivo* environment as closely as possible, bioreactors can enhance the formation of functional tissues. Bioreactors can induce shear stress and hydrostatic pressure needed for the formation of certain types of tissues such as cartilage and bone.^317–319^ However, despite the progress made in building tissues, further optimization is required to produce tissues in a timely manner and reach the proper differentiation state of cells needed for clinical use.

Recently, multiphoton ablation,^320^ 3D bioprinting,^321,322^ and microchamber-based approaches^323^ have emerged as promising technologies for tissue engineering. Multiphoton ablation, a technique that uses laser light to selectively remove material from a sample in a highly precise manner, has been used to create channels of specific size and shape within a scaffold material such as collagen. The scaffold can then be seeded with ECs to create functional BVs.^320^ The use of pre-formed microvessels as a guide for endothelial cell growth can help ensure that the resulting vessels are properly aligned and connected, which is important for their proper function. 3D Bioprinting technology has the potential to revolutionize tissue engineering and regenerative medicine. By precisely depositing cells and biomaterials in a controlled manner, it is possible to create complex, 3D structures that mimic the architecture and function of natural tissues and organs. This has opened up new avenues for the formation of functional organs and large-scale growth of artificial tissues.^79^ Bioprinting of organoid-derived stem cells into ECM matrices has been shown to promote self-organization of cells into tissue-like structures, which could be used for drug discovery, disease modeling, and regeneration. Moreover, the recent development of multicell-type bioprinted lumenized intestinal epithelium is a promising step toward creating functional tissues for transplantation.^79^ Microchamber-based approaches, on the other hand, allow for the formation of organoids with defined cell numbers and spatial arrangements, which can be further assembled into larger structures. These technologies offer new opportunities for the development of effective tissue engineering approaches for regenerative medicine.

*In vivo* engraftment approaches provide important validation for the functionality and regenerative potential of *in vitro* engineered tissues. They demonstrate that these tissues can integrate with the surrounding tissue, support vascularization, and perform their intended functions. For instance, kidney organoid transplantation under the kidney capsule of immunodeficient mice has allowed mouse ECs to infiltrate the transplanted kidney organoid and promote vascularization, urine production, and renal recovery.^21,324–326^ Also, cardiac tissue engineering has been applied using human embryonic stem cell-derived ECs, generating *in vitro* perfusable microvessels. When implanted on infarcted rat hearts, the microvessels are able to be engrafted with rat cardiac vasculature being fully functional.^327^ Tissue engineering techniques also have the potential to pave the way for future regenerative medicine therapies, where *in vitro* engineered tissues could be transplanted into patients to treat a variety of diseases and injuries. However, more research is needed to further improve the functionality, safety, and long-term viability of these engineered tissues before they can be applied in a clinical setting.

## V. CONCLUSIONS, CURRENT CHALLENGES, AND FUTURE PERSPECTIVES

Microfluidic-based cell culture technologies offer a high degree of control on the experimental design, system parameters, and system modeling characterization. Thus, they hold great promise for the development of relevant preclinical models (OoC and organoids), drug discovery, as well as personalized and regenerative medicine. In this scenario, the implementation of fluid flow in 3D perfusable microfluidic devices is fundamental to mimic human physiopathology in many organs. Though several vascularized organ 3D models have already been developed,^17,152,307,328^ current challenges consist of reproducing the complex organ's microenvironment and vessel complex geometry considering the strong coupling between biology and mechanics. Despite the fact that the implementation of 3D organ vascularization is progressing, lymphatic vasculature is lacking from many current applications and should be considered due to its crucial physiological role. Another challenge is the *in situ* analysis of the perfused microfluidic devices at the transcriptome and metabolome level (key for translational applications) due to the low volume of cells and secreted metabolites in the chips. For that, the use of biosensors incorporating real-time monitoring is essential, as well as the methods to scale up properly designed 3D cell growing to allow the implementation of high-throughput methods (such as single cell RNA sequencing). In addition, the choice of defined perfusate together with human-biocompatible derived matrices is to be considered.

Despite the existing limitations, OoC and organoids are already being used for drug discovery studies, and show significant potential for personalized treatment decisions in diseases like cancer. The above-mentioned progressing construction of tissues vascularization carry high expectations for subsequent transplantation of large-scale prevascularized constructs in regenerative medicine. These constructs would enhance grafts viability and would favor host tissue regeneration.

Precise microvascular network can be provided with 3D bioprinting technology,^77–79,329^ a field in expansion, which will allow, together with fluid flow, the formation of functional organs and long-term and large-scale growth of artificial tissues. Engineering strategies combined with computational models for biophysical and topological parameters will help in reducing variability, while increasing the degree of automation. Especially important will be future implementation of engineering personalized “humans-on-chips” in which several organs derived from a patient are integrated in the presence of vascular, immune, and microbiome compartments. Altogether, bringing accurately perfused vascular biology to bioengineering approaches is necessary to advance drug discovery and personalized and regenerative medicine in which the collaboration between engineers, clinicians, chemists and biologists is fundamental.

## SUPPLEMENTARY MATERIAL

See the supplementary material for Fig. 1, which shows the evolution of 3D cell culture and perfused 3D cell culture tendencies in publications for the last 30 years. Specifically, the percentage of cell culture publications including 3D ECM and perfused 3D ECM terms is analyzed, as well as the fold increase of publications including 3D ECM and perfused 3D ECM terms (compared with 1993–2002 decade), and the percentage of 3D ECM cell culture publications that include fluid flow term.

## Data Availability

Data sharing is not applicable to this article as no new data were created or analyzed in this study.
